# P02.155. Stress Management and Resilience Training (SMART) program to decrease stress and enhance resilience among breast cancer survivors: a randomized trial

**DOI:** 10.1186/1472-6882-12-S1-P211

**Published:** 2012-06-12

**Authors:** A Sood, C Loprinzi, V Sharma, K Prasad

**Affiliations:** 1 Mayo Clinic Rochester, Rochester, USA

## Purpose

Patients with breast cancer experience considerable stress and anxiety related to their diagnosis, with resulting lower quality of life. The current study was designed to assess the effect of a Stress Management and Resiliency Training (SMART) program for increasing resiliency and decreasing stress and anxiety among a group of mentors who themselves previously were diagnosed with breast cancer (pink ribbon mentors).

## Methods

Twenty-five pink ribbon mentors at Mayo Clinic Rochester were randomized in a single-blind wait-list controlled clinical trial to either the SMART intervention or a control group for twelve weeks. The intervention involved two 90 minute group training sessions followed by a brief individual session and four weekly telephone calls. After the initial group session, all participants in the intervention arm were provided with a DVD to practice paced breathing meditation. Primary outcome measures assessed at baseline and week 12 included the Connor Davidson Resilience Scale (CDRS), Perceived Stress Scale (PSS), Smith Anxiety Scale (SAS), and Linear Analog Self Assessment Scale (LASA).

## Results

Twenty patients completed the study. A statistically significant improvement in resilience, perceived stress, anxiety and overall quality of life at 12 weeks, compared to baseline, was observed in the study arm (CDRS: 73.6±10.1 vs. 81.3±9.1, p=0.010), (PSS: 22.1±5.9 vs. 12.8±6.6, p=0.003), (SAS: 49.4±18.2 vs. 33.3±11.7, p=0.002) and LASA (38.4±6.1 vs. 44.5±3.5, p=0.002). No significant difference in the any of these measures was noted in the control group.

## Conclusion

This study demonstrates that a brief, predominantly group-based resilience training intervention is feasible in patients with previous breast cancer. Further, the intervention provided statistically significant and clinically meaningful improvement on resilience, stress, anxiety and overall quality of life. Future larger clinical trials with this intervention are warranted.

